# Bis(1*H*-benzimidazole-κ*N* ^3^)bis­(4-methyl­benzoato-κ^2^ *O*,*O*′)copper(II)

**DOI:** 10.1107/S1600536808012440

**Published:** 2008-05-03

**Authors:** Wen-Dong Song, Xiang-Hu Huang, Hui Wang

**Affiliations:** a College of Science, Guang Dong Ocean University, Zhan Jiang 524088, People’s Republic of China; b College of Fisheries, Guang Dong Ocean University, Zhan Jiang 524088, People’s Republic of China

## Abstract

In the title mononuclear complex, [Cu(C_8_H_7_O_2_)_2_(C_7_H_6_N_2_)_2_], the Cu^II^ atom lies on an inversion centre and is coordinated by two O atoms of two monodentate 4-methyl­benzoate ligands and two N atoms of two benzimidazole ligands in a square-planar geometry. The mol­ecules are linked into chains running parallel to the *b* axis by inter­molecular N—H⋯O hydrogen bonds and by π–π stacking inter­actions [centroid–centroid distance = 3.669 (2) Å] involving centrosymmetrically related imidazole rings.

## Related literature

For related literature, see: Song *et al.* (2007 ▶).
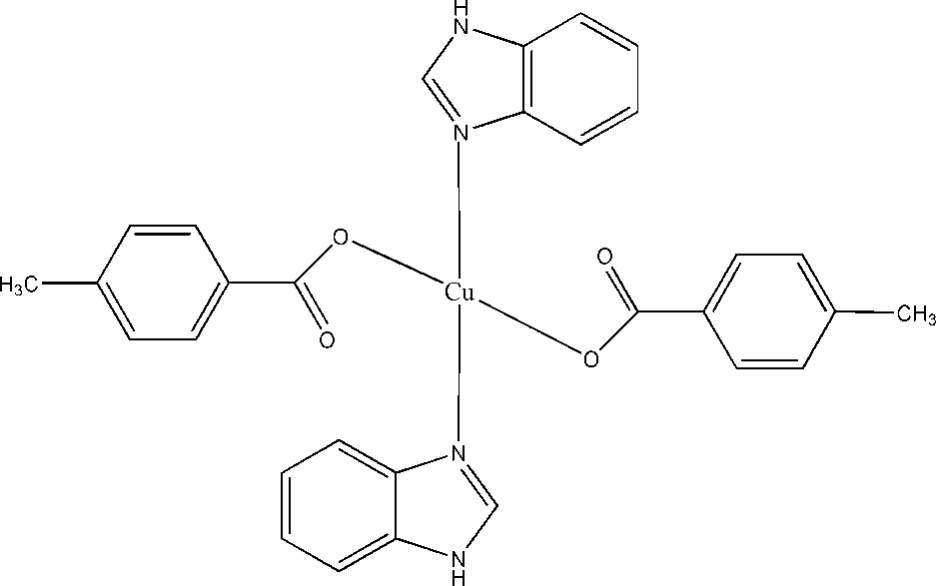


## Experimental

### 

#### Crystal data

[Cu(C_8_H_7_O_2_)_2_(C_7_H_6_N_2_)_2_]
*M* *_r_* = 570.10
Triclinic, 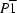

*a* = 7.2623 (2) Å
*b* = 7.6068 (1) Å
*c* = 12.9624 (2) Å
α = 99.687 (2)°
β = 96.390 (1)°
γ = 104.776 (3)°
*V* = 673.54 (3) Å^3^
*Z* = 1
Mo *K*α radiation
μ = 0.85 mm^−1^
*T* = 296 (2) K
0.40 × 0.30 × 0.20 mm

#### Data collection

Bruker APEXII area-detector diffractometer
Absorption correction: multi-scan (*SADABS*; Sheldrick, 1996 ▶) *T*_min_ = 0.726, *T*_max_ = 0.848
8200 measured reflections
2743 independent reflections
2445 reflections with *I* > 2σ(*I*)
*R*_int_ = 0.026

#### Refinement

*R*[*F* ^2^ > 2σ(*F* ^2^)] = 0.035
*wR*(*F* ^2^) = 0.121
*S* = 0.88
2743 reflections
179 parameters
H-atom parameters constrained
Δρ_max_ = 0.31 e Å^−3^
Δρ_min_ = −0.22 e Å^−3^

### 

Data collection: *APEX2* (Bruker, 2004 ▶); cell refinement: *SAINT* (Bruker, 2004 ▶); data reduction: *SAINT*; program(s) used to solve structure: *SHELXS97* (Sheldrick, 2008 ▶); program(s) used to refine structure: *SHELXL97* (Sheldrick, 2008 ▶); molecular graphics: *SHELXTL* (Sheldrick, 2008 ▶); software used to prepare material for publication: *SHELXTL*.

## Supplementary Material

Crystal structure: contains datablocks I, global. DOI: 10.1107/S1600536808012440/rz2208sup1.cif

Structure factors: contains datablocks I. DOI: 10.1107/S1600536808012440/rz2208Isup2.hkl

Additional supplementary materials:  crystallographic information; 3D view; checkCIF report

## Figures and Tables

**Table 1:** Hydrogen-bond geometry (Å, °)

*D*—H⋯*A*	*D*—H	H⋯*A*	*D*⋯*A*	*D*—H⋯*A*
N2—H2⋯O2^i^	0.86	1.95	2.780 (2)	163

Symmetry code: (i) 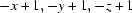.

**Fig. 1.**
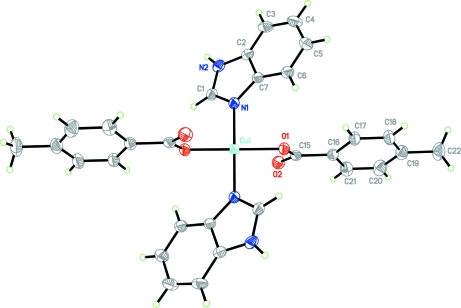
The molecular structure of the title compound, showing the atom-numbering scheme and 30% probability displacement ellipsoids. Unlabelled atoms are related to the labelled atoms by the symmetry operator (1-x, 2-y, 1-z).

**Fig. 2.**
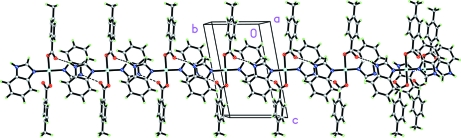
Packing diagram of the title compound viewed approximately along the a axis. Dashed lines indicate hydrogen bonds.

## supplementary crystallographic information

### Comment

In the structural investigation of 4-methylbenzate complexes, it has been found that 4-methylbenzoic acid can act as a multidentate ligand (Song *et al.*, 2007), with versatile binding and coordination modes. In this paper, we report the crystal structure of the title compound, a new Cu complex obtained by the reaction of 4-methylbenzoic acid, benzimidazole and copper chloride in alkaline aqueous solution.

As illustrated in Fig. 1, the complex molecule has an inversion symmetry where the Cu^II^ atom exists in a square planar coordination geometry, defined by two carboxyl O atoms from two monodentate 4-methylbenzate ligands and two N atoms from two benzimidazole ligands. In the crystal structure, intermolecular N—H···O hydrogen bonding interactions (Table 1) and π-π stacking interactions (centroid-centroid distance = 3.669 (2) Å) occurring between the imidazole rings of centrosymmetrically-related complexes form chains running parallel to the b axis (Fig. 2).

### Experimental

A mixture of copper chloride (1 mmol), 4-methylbenzoic acid (1 mmol), benzimidazole (1 mmol), NaOH (1.5 mmol) and H_2_O (12 ml) was placed in a 23 ml Teflon reactor and heated to 433 K for three days. After cooling to room temperature at a rate of 10 K h^-1^, the crystals obtained were washed with water and dryed in air.

### Refinement

All H atoms were placed at calculated positions and treated as riding on their parent atoms, with C—H = 0.93 - 0.96 Å, N—H = 0.86 Å, and with *U*_iso_(H) = 1.2 *U*_eq_(C, N) or 1.5 *U*_eq_(C) for methyl H atoms.

### Crystal data

**Table:**

[Cu(C_8_H_7_O_2_)_2_(C_7_H_6_N_2_)_2_]	*Z* = 1
*M**_r_* = 570.10	*F*_000_ = 295
Triclinic, *P*1	*D*_x_ = 1.406 Mg m^−^^3^
Hall symbol: -P 1	Mo *K*α radiation λ = 0.71073 Å
*a* = 7.2623 (2) Å	Cell parameters from 3600 reflections
*b* = 7.6068 (1) Å	θ = 1.4–28º
*c* = 12.9624 (2) Å	µ = 0.85 mm^−^^1^
α = 99.687 (2)º	*T* = 296 (2) K
β = 96.390 (1)º	Block, blue
γ = 104.776 (3)º	0.40 × 0.30 × 0.20 mm
*V* = 673.54 (3) Å^3^	

### Data collection

**Table:**

Bruker APEXII area-detector diffractometer	2743 independent reflections
Radiation source: fine-focus sealed tube	2445 reflections with *I* > 2σ(*I*)
Monochromator: graphite	*R*_int_ = 0.026
*T* = 296(2) K	θ_max_ = 26.5º
φ and ω scans	θ_min_ = 1.6º
Absorption correction: multi-scan(SADABS; Sheldrick, 1996)	*h* = −9→9
*T*_min_ = 0.726, *T*_max_ = 0.848	*k* = −9→9
8200 measured reflections	*l* = −16→16

### Refinement

**Table:**

Refinement on *F*^2^	Secondary atom site location: difference Fourier map
Least-squares matrix: full	Hydrogen site location: inferred from neighbouring sites
*R*[*F*^2^ > 2σ(*F*^2^)] = 0.035	H-atom parameters constrained
*wR*(*F*^2^) = 0.121	*w* = 1/[σ^2^(*F*_o_^2^) + (0.1115*P*)^2^] where *P* = (*F*_o_^2^ + 2*F*_c_^2^)/3
*S* = 0.88	(Δ/σ)_max_ = 0.045
2743 reflections	Δρ_max_ = 0.31 e Å^−^^3^
179 parameters	Δρ_min_ = −0.22 e Å^−^^3^
Primary atom site location: structure-invariant direct methods	Extinction correction: none

### Special details

**Table:**

Geometry. All e.s.d.'s (except the e.s.d. in the dihedral angle between two l.s. planes) are estimated using the full covariance matrix. The cell e.s.d.'s are taken into account individually in the estimation of e.s.d.'s in distances, angles and torsion angles; correlations between e.s.d.'s in cell parameters are only used when they are defined by crystal symmetry. An approximate (isotropic) treatment of cell e.s.d.'s is used for estimating e.s.d.'s involving l.s. planes.
Refinement. Refinement of *F*^2^ against ALL reflections. The weighted *R*-factor *wR* and goodness of fit *S* are based on *F*^2^, conventional *R*-factors *R* are based on *F*, with *F* set to zero for negative *F*^2^. The threshold expression of *F*^2^ > σ(*F*^2^) is used only for calculating *R*-factors(gt) *etc*. and is not relevant to the choice of reflections for refinement. *R*-factors based on *F*^2^ are statistically about twice as large as those based on *F*, and *R*- factors based on ALL data will be even larger.

### Fractional atomic coordinates and isotropic or equivalent isotropic displacement parameters (Å^2^)

**Table:**

	*x*	*y*	*z*	*U*_iso_*/*U*_eq_	
C1	0.3775 (3)	0.6448 (3)	0.56672 (17)	0.0404 (5)	
H1	0.4583	0.6988	0.6312	0.048*	
C2	0.1775 (3)	0.4328 (3)	0.43989 (18)	0.0397 (5)	
C3	0.0533 (4)	0.2729 (3)	0.3739 (2)	0.0516 (6)	
H3	0.0277	0.1586	0.3942	0.062*	
C4	−0.0289 (4)	0.2922 (4)	0.2778 (2)	0.0602 (7)	
H4	−0.1113	0.1882	0.2313	0.072*	
C5	0.0079 (4)	0.4657 (4)	0.2474 (2)	0.0553 (7)	
H5	−0.0517	0.4739	0.1818	0.066*	
C6	0.1304 (3)	0.6235 (3)	0.31304 (18)	0.0449 (5)	
H6	0.1530	0.7380	0.2931	0.054*	
C7	0.2189 (3)	0.6062 (3)	0.40969 (17)	0.0355 (4)	
C15	0.5949 (3)	0.8828 (3)	0.31242 (16)	0.0400 (5)	
C16	0.5876 (4)	0.8343 (3)	0.19460 (17)	0.0404 (5)	
C17	0.4260 (4)	0.8288 (3)	0.12438 (18)	0.0462 (5)	
H17	0.3205	0.8574	0.1503	0.055*	
C18	0.4215 (4)	0.7808 (4)	0.01597 (18)	0.0553 (7)	
H18	0.3117	0.7766	−0.0299	0.066*	
C19	0.5742 (5)	0.7394 (3)	−0.02547 (19)	0.0548 (7)	
C20	0.7351 (5)	0.7454 (4)	0.0439 (2)	0.0631 (8)	
H20	0.8405	0.7179	0.0172	0.076*	
C21	0.7426 (4)	0.7920 (4)	0.1535 (2)	0.0547 (6)	
H21	0.8522	0.7947	0.1990	0.066*	
C22	0.5672 (6)	0.6885 (4)	−0.1443 (2)	0.0736 (9)	
H22A	0.4408	0.6111	−0.1768	0.110*	
H22B	0.6610	0.6226	−0.1589	0.110*	
H22C	0.5955	0.7996	−0.1725	0.110*	
Cu1	0.5000	1.0000	0.5000	0.03466 (16)	
N1	0.3479 (3)	0.7375 (2)	0.49250 (13)	0.0363 (4)	
N2	0.2793 (3)	0.4642 (3)	0.54000 (15)	0.0423 (4)	
H2	0.2800	0.3829	0.5787	0.051*	
O1	0.4718 (2)	0.9625 (2)	0.34485 (11)	0.0403 (4)	
O2	0.7185 (3)	0.8455 (2)	0.37312 (13)	0.0503 (4)	

### Atomic displacement parameters (Å^2^)

**Table:**

	*U*^11^	*U*^22^	*U*^33^	*U*^12^	*U*^13^	*U*^23^
C1	0.0484 (12)	0.0394 (11)	0.0344 (10)	0.0100 (9)	0.0095 (9)	0.0123 (9)
C2	0.0398 (11)	0.0345 (11)	0.0472 (12)	0.0092 (9)	0.0146 (9)	0.0120 (9)
C3	0.0490 (13)	0.0304 (11)	0.0703 (17)	0.0020 (10)	0.0139 (12)	0.0077 (11)
C4	0.0503 (14)	0.0473 (14)	0.0661 (17)	−0.0060 (12)	0.0027 (12)	0.0001 (12)
C5	0.0482 (13)	0.0559 (15)	0.0496 (14)	0.0006 (12)	−0.0038 (10)	0.0070 (11)
C6	0.0436 (12)	0.0433 (12)	0.0450 (12)	0.0051 (10)	0.0048 (9)	0.0134 (10)
C7	0.0347 (10)	0.0322 (10)	0.0387 (10)	0.0064 (8)	0.0083 (8)	0.0077 (8)
C15	0.0504 (12)	0.0264 (10)	0.0361 (11)	−0.0028 (9)	0.0011 (9)	0.0122 (8)
C16	0.0547 (13)	0.0270 (10)	0.0367 (11)	0.0063 (9)	0.0049 (9)	0.0081 (8)
C17	0.0549 (13)	0.0463 (13)	0.0353 (11)	0.0118 (11)	0.0060 (9)	0.0068 (9)
C18	0.0672 (16)	0.0571 (14)	0.0345 (11)	0.0113 (13)	0.0016 (11)	0.0039 (11)
C19	0.0819 (19)	0.0394 (12)	0.0422 (13)	0.0155 (12)	0.0152 (12)	0.0046 (10)
C20	0.079 (2)	0.0590 (17)	0.0591 (16)	0.0291 (15)	0.0263 (15)	0.0094 (13)
C21	0.0649 (16)	0.0508 (14)	0.0498 (14)	0.0200 (13)	0.0067 (12)	0.0100 (11)
C22	0.114 (3)	0.0659 (18)	0.0417 (14)	0.0259 (18)	0.0221 (15)	0.0049 (13)
Cu1	0.0445 (2)	0.0294 (2)	0.0273 (2)	0.00483 (15)	0.00285 (14)	0.00858 (14)
N1	0.0443 (10)	0.0329 (9)	0.0307 (8)	0.0074 (8)	0.0053 (7)	0.0091 (7)
N2	0.0533 (11)	0.0344 (9)	0.0461 (10)	0.0143 (8)	0.0149 (8)	0.0197 (8)
O1	0.0508 (9)	0.0359 (8)	0.0310 (7)	0.0067 (7)	0.0045 (6)	0.0076 (6)
O2	0.0587 (10)	0.0457 (9)	0.0455 (9)	0.0101 (8)	−0.0018 (7)	0.0202 (7)

### Geometric parameters (Å, °)

**Table:**

C1—N1	1.315 (3)	C16—C17	1.390 (3)
C1—N2	1.342 (3)	C17—C18	1.386 (3)
C1—H1	0.9300	C17—H17	0.9300
C2—N2	1.371 (3)	C18—C19	1.368 (4)
C2—C3	1.394 (3)	C18—H18	0.9300
C2—C7	1.407 (3)	C19—C20	1.379 (4)
C3—C4	1.368 (4)	C19—C22	1.516 (3)
C3—H3	0.9300	C20—C21	1.396 (4)
C4—C5	1.410 (4)	C20—H20	0.9300
C4—H4	0.9300	C21—H21	0.9300
C5—C6	1.378 (3)	C22—H22A	0.9600
C5—H5	0.9300	C22—H22B	0.9600
C6—C7	1.386 (3)	C22—H22C	0.9600
C6—H6	0.9300	Cu1—O1^i^	1.9630 (14)
C7—N1	1.402 (3)	Cu1—O1	1.9630 (14)
C15—O2	1.246 (3)	Cu1—N1	2.0007 (16)
C15—O1	1.272 (3)	Cu1—N1^i^	2.0007 (16)
C15—C16	1.501 (3)	N2—H2	0.8600
C16—C21	1.384 (4)		
			
N1—C1—N2	113.20 (19)	C19—C18—H18	119.1
N1—C1—H1	123.4	C17—C18—H18	119.1
N2—C1—H1	123.4	C18—C19—C20	118.2 (2)
N2—C2—C3	132.2 (2)	C18—C19—C22	121.0 (3)
N2—C2—C7	105.66 (19)	C20—C19—C22	120.8 (3)
C3—C2—C7	122.2 (2)	C19—C20—C21	121.2 (3)
C4—C3—C2	116.8 (2)	C19—C20—H20	119.4
C4—C3—H3	121.6	C21—C20—H20	119.4
C2—C3—H3	121.6	C16—C21—C20	120.2 (3)
C3—C4—C5	121.7 (2)	C16—C21—H21	119.9
C3—C4—H4	119.2	C20—C21—H21	119.9
C5—C4—H4	119.2	C19—C22—H22A	109.5
C6—C5—C4	121.3 (2)	C19—C22—H22B	109.5
C6—C5—H5	119.3	H22A—C22—H22B	109.5
C4—C5—H5	119.3	C19—C22—H22C	109.5
C5—C6—C7	117.9 (2)	H22A—C22—H22C	109.5
C5—C6—H6	121.0	H22B—C22—H22C	109.5
C7—C6—H6	121.0	O1^i^—Cu1—O1	180.00 (10)
C6—C7—N1	131.56 (19)	O1^i^—Cu1—N1	88.20 (6)
C6—C7—C2	120.1 (2)	O1—Cu1—N1	91.80 (6)
N1—C7—C2	108.32 (19)	O1^i^—Cu1—N1^i^	91.80 (6)
O2—C15—O1	123.3 (2)	O1—Cu1—N1^i^	88.20 (6)
O2—C15—C16	119.9 (2)	N1—Cu1—N1^i^	180.00 (11)
O1—C15—C16	116.81 (19)	C1—N1—C7	105.17 (17)
C21—C16—C17	118.4 (2)	C1—N1—Cu1	122.68 (15)
C21—C16—C15	120.2 (2)	C7—N1—Cu1	131.45 (14)
C17—C16—C15	121.3 (2)	C1—N2—C2	107.64 (18)
C18—C17—C16	120.3 (2)	C1—N2—H2	126.2
C18—C17—H17	119.9	C2—N2—H2	126.2
C16—C17—H17	119.9	C15—O1—Cu1	109.52 (13)
C19—C18—C17	121.8 (3)		

Symmetry codes: (i) −*x*+1, −*y*+2, −*z*+1.

### Hydrogen-bond geometry (Å, °)

**Table:**

*D*—H···*A*	*D*—H	H···*A*	*D*···*A*	*D*—H···*A*
N2—H2···O2^ii^	0.86	1.95	2.780 (2)	163

Symmetry codes: (ii) −*x*+1, −*y*+1, −*z*+1.
